# Characteristics and factors influencing hypothalamic pituitary dysfunction in patients with craniopharyngioma

**DOI:** 10.3389/fendo.2023.1180591

**Published:** 2023-06-02

**Authors:** Ying Guo, Lili Pei, Yuzheng Li, Chunde Li, Songbai Gui, Ming Ni, Pinan Liu, Yazhuo Zhang, Liyong Zhong

**Affiliations:** ^1^ Department of Endocrinology, Beijing Tiantan Hospital, Capital Medical University, Beijing, China; ^2^ Department of Neurosurgery, Beijing Tiantan Hospital, Capital Medical University, Beijing, China; ^3^ Beijing Neurosurgical Institute, Capital Medical University, Beijing, China

**Keywords:** clinical prognosis, hypothalamic syndrome, endocrine function, papillary craniopharyngioma, adamantinomatous craniopharyngioma

## Abstract

**Background:**

Craniopharyngioma is a benign tumor originating from the sellar region. Damages in this area caused by the tumor itself, surgery, or radiotherapy may result in severe hypothalamic-pituitary dysfunction (HPD) and eventually lead to a significant impairment in the long-term quality of life of patients. This study aimed to investigate the characteristics of HPD in patients with adamantinomatous craniopharyngioma (ACP) or papillary craniopharyngioma (PCP) and to identify the factors affecting HPD after surgery.

**Methods:**

In this single-center retrospective study, a total of 742 patients with craniopharyngioma were included. The neuroendocrine function of these patients before and after surgery was investigated. The differences in hypothalamic-pituitary function between the ACP and PCP groups were compared. The factors influencing the aggravation of HPD after surgery were identified.

**Results:**

The median follow-up after surgery was 15 months. Before surgery, the proportion of patients with diabetes insipidus (DI) and hyperprolactinemia in the PCP group was significantly higher than that in the ACP group (*P*<0.01), and the proportion of patients with adrenocortical hypofunction in the PCP group was significantly lower than that in the ACP group (*P*=0.03). Most cases of ACP originated in the sellar region, while most cases of PCP originated in the suprasellar region (*P*<0.01). More patients experienced adenohypophyseal hypofunction, DI, and hypothalamic obesity at postoperative follow-up than at onset in both the ACP and PCP groups (both *P*<0.01), with a higher increase observed in the ACP group (*P*<0.01). Older age at CP onset, tumor recurrence or progression, and ACP type were risk factors for postoperative aggravation of HPD in CP patients.

**Conclusion:**

Surgical treatment significantly aggravated HPD in both the ACP and PCP groups, but the specific characteristics and risk factors leading to aggravation were different between the two groups.

## 1 Introduction

Craniopharyngioma (CP) is a benign tumor of the central nervous system located in the sellar or parasellar region. According to the 2021 World Health Organization Classification of Tumors of the Central Nervous System, there are two types of CP, adamantinomatous craniopharyngioma (ACP) and papillary craniopharyngioma (PCP), with significantly different etiologies, imaging features, histopathological characteristics, genetic changes, and methylation profiles (1). ACP is derived from the epithelial cells of the craniopharyngeal canal or residual tissues of Rathke’s pouch, while PCP originates from the residual squamous cells of the original oral fossa (2). CP accounts for approximately 2–3% of all brain tumors in adults and 6–10% in children. It is often accompanied by tumor mass effects, such as headaches, visual field impairment, adenohypophyseal dysfunction, and hypothalamic syndrome (3–5). Destruction of the hypothalamic-pituitary region by tumor tissues or by damages from surgery or radiotherapy aggravates hypothalamic-pituitary dysfunction (HPD) in patients with CP, leading to a reduced quality of life and decreased survival rate (3, 6).

The high heterogeneity of hypothalamic-pituitary dysfunction in patients with CP is caused by multiple factors, such as age of onset, pathological type, tumor size and location, treatment method, and recurrence (7–9). However, no large-scale clinical studies have investigated the characteristics, differences, and influencing factors of HPD in patients with ACP and PCP. Therefore, in the present study, we collected the clinical data of a large group of patients with CP, aiming to summarize the characteristics of HPD in patients with ACP or PCP. The neuroendocrine function of these patients before and after surgery was compared and the factors influencing the aggravation of HPD after surgery were identified.

## 2 Subjects and methods

### 2.1 Study population

**Figure 1:**
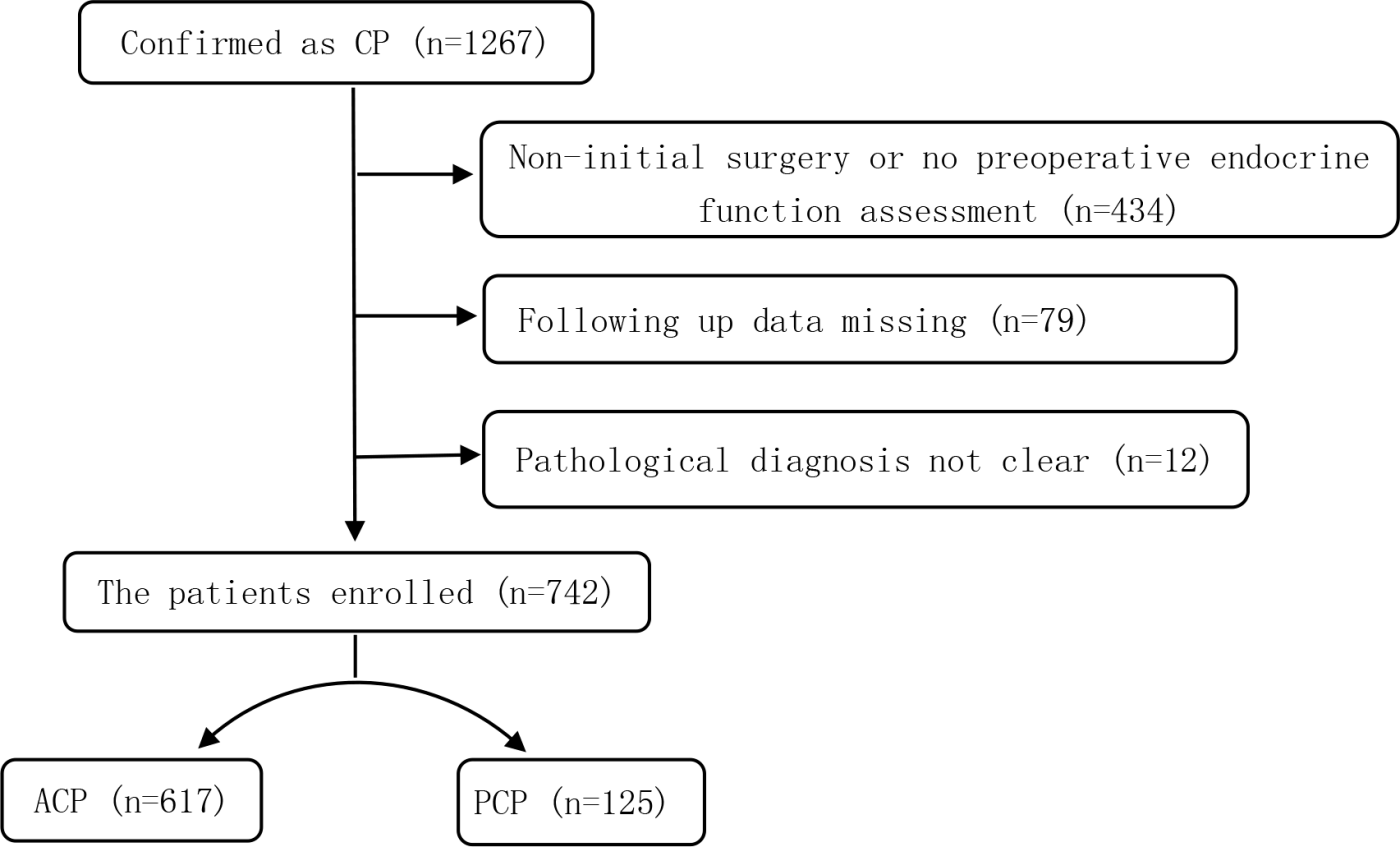
Flowchart of the patient enrollment process.

In this single-center retrospective study, a total of 1,267 CP patients who were hospitalized at the Beijing Tiantan Hospital between January 2015 and September 2019 were screened. The inclusion criteria were as follows (1): the primary surgical resection was performed in our hospital; (2) the data on the preoperative and postoperative (more than 3 months after surgery) endocrine function could be obtained; (3) ACP or PCP was pathologically diagnosed. Subjects were excluded from this study if they (1) had other diseases in the saddle region; (2) had dysfunction of the hypothalamic-pituitary target gland axes due to other primary or secondary diseases; or (3) were on hormonal therapies for other reasons. After applying the inclusion and exclusion criteria, a total of 742 patients with CP were enrolled (**Figure 1**).

### 2.2 Methods

The baseline characteristics (e.g., age of onset, sex, tumor size, tumor location, and pathological type), operation-related indicators [e.g., surgical method, gross-total resection (GTR) or partial resection], and hypothalamic-pituitary functions in all patients before and after surgery were collected. During surgery, Sharp dissection is typically used to separate the tumor from the stalk while preserving the stalk’s integrity as much as possible. Following surgery, CP patients were monitored using imaging at 3 days, 3 months, 6 months, and 1 year, with annual reviews thereafter. Radiotherapy for suitable CP patients was performed using Leksell Gamma Knife radiation technology. The neuroendocrine dysfunction scale (NEDS) was used to evaluate the degree of HPD. The risk factors for postoperative aggravation of HPD were identified by univariate and multivariate logistic regression analyses.

The NEDS evaluates 5 hypothalamic syndrome disorders and 5 adenohypophyseal dysfunction disorders. The diagnosis of each disorder (shown as each index) was in accordance with corresponding guidelines or medical consensus. For each index, no points were assigned for normal function, while 1 point was assigned for dysfunction. The scores of the 10 indices were added to obtain a total score, which was used to indicate the degree of HPD in patients with CP (**Table 1**).

**Table 1:** The NEDS for the evaluation of HPD in CP patients.

Indices	Diagnostic criteria	Score
Hypothalamic syndrome		
ABTR	Persistent body temperature >37.3°C or <35.0°C, without infection or other diseases that cause abnormal body temperature	1
HO	For children or adolescents who are under 18 years of age, a BMI greater than or equal to the corresponding sex, age group 95^th^ percentile cut-off point, according to the screening guidelines for overweight and obesity among Chinese school-age children and adolescents (10). For adults over 18 years of age, BMI ‗28 kg/m^2^.	1
Sleep disorder	For children and adolescents under 18 years of age, sleep disorder was diagnosed according to the Sleep Disturbance Scale for Children (SDSC) (11). For adults over 18 years of age, sleep disorder was diagnosed using the Pittsburgh Sleep Quality Index (PSQI): >5, poor sleep quality; ≤5, good quality (12).	1
PCA	PCA was diagnosed according to the Eysenck Personality Questionnaire-Brief Version (13), the Wechsler Intelligence Scale, 4th edition (WIS-IV), and the Full-Scale IQ (FSIQ) test (14).	1
DI	Urine volume >50 mL/kg/24h or >4000 mL/24h; urine specific gravity <1.005 (15).	1
Adenohypophyseal dysfunction (4, 16, 17)		
HPA	Based on the absence of glucocorticoid drug usage and the presence of clinical symptoms such as fatigue, anorexia, and hyponatremia, etc., the patients’ serum concentrations of COR and ACTH concentrations should meet one of the following two criteria: 1. Serum concentrations of COR <5 μg/dL and ACTH <25.0 pmol/L at 8 am on two separate occasions, 2. COR <5 μg/dL and ACTH <25.0 pmol/L at 8 am once, with disappearance of COR/ACTH circadian rhythm. ^a^	1
HPT	FT4 is lower than the reference range, accompanied by normal, decreased, or slightly increased TSH levels (<10 mU/L) twice, or once combined with symptoms and signs of hypothyroidism.	1
HPG ^b^	Men: decreased T levels; decreased FSH and LH levels or levels at the lower limit of normal values. Premenopausal women: decreased E2 levels; decreased FSH and LH levels or levels at the lower limit of normal values. Postmenopausal women: decreased E2 levels; no increase in FSH and LH levels.	1
GHD	For patients under 18 years of age, the diagnostic criteria of GHD in children were used ^c^. For patients aged 18 years or older, the diagnostic criteria of GHD in adults was used ^d^.	1
Hyperprolactinemia	Serum prolactin levels above the normal range (>20 ng/mL in men and postmenopausal women, >30 ng/mL in premenopausal women)	1

a Disappearance of COR/ACTH circadian rhythm: Serum concentrations of 8 am COR < 5 μg/dL and ACTH < 25.0 pmol/L, 4 pm COR < 2.5 μg/dL and ACTH < 15.0 pmol/L, 0 am COR < 2.5 μg/dL and ACTH < 15.0 pmol/L.

b HPG was not assessed in males under 14 years and females under 13 years.

c 1. Height less than -2SD: IGF-1 and IGFBP-3 are below the normal range for age and sex matching controls. 2. Height from 0 to -2SD: IGF-1 and IGFBP-3 are below the normal range for age and sex matching controls, accompanied by at least one other adenohypophysis hypofunction or at least one GH stimulation test (exercise, sleep, arginine, insulin) with a GH peak < 5 μg/L.

d IGF-1 and IGFBP-3 are below the normal range for age and sex matching controls, accompanied by at least one other adenohypophysis hypofunction or at least one of the GH stimulation tests with the peak value of GH < 5 μg/L.

ABTR, abnormal body temperature regulation; ACTH, adrenocorticotrophic hormone; BMI, body mass index; DI, diabetes insipidus; COR, cortisol; E2, estradiol; FSH, follicle-stimulating hormone; FT4, free thyroxine; GH, growth hormone; GHD, growth hormone deficiency; HPA, hypothalamus-pituitary-adrenal axis; HPG, hypothalamus-pituitary-gonad axis; HPT, hypothalamus-pituitary-thyroid axis; HO, hypothalamic obesity; IGF-1, insulin-like growth factor-1; IGFBP-3, insulin-like growth factor-binding protein 3; LH, luteinizing hormone; PCA, personality and cognitive abnormality; T, testosterone; TSH, thyroid stimulating hormone.

### 2.3 Data analysis

Measurement data conforming to a normal distribution were presented as mean ± standard deviation (SD) and compared using Student’s *t*-test. Non-normally distributed data were presented as median (interquartile range) and compared using the Mann-Whitney U test. Categorical data were presented as frequency (n) and percentage (%) and compared by the Chi-squared test. Univariate and multivariate logistic regression analyses were used to identify influencing factors for the aggravation of endocrine dysfunction. A *P*-value of less than 0.05 indicated statistical significance unless stated otherwise. Data were analyzed using the SPSS Statistics 23 software (SPSS Inc.).

## 3 Results

### 3.1 Baseline data and operation-related indicators

**Figure 2:**
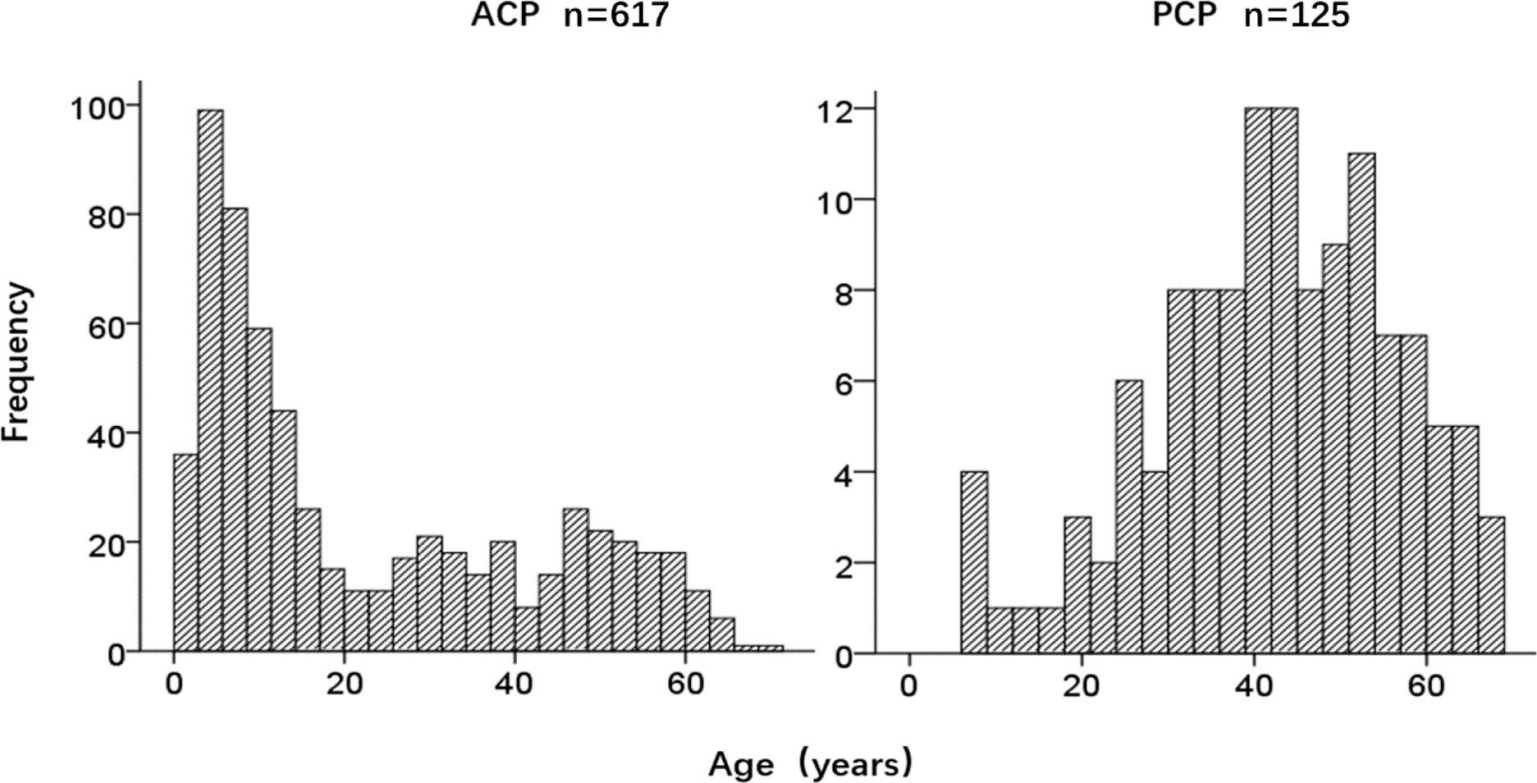
Age Distribution in the ACP and PCP groups.

A total of 742 CP patients were enrolled in this study, including 617 cases with ACP and 125 cases with PCP, with a ratio of 4.9:1. There were 421 males and 321 females, with a male to female (M/F) ratio of 1.3:1. The M/F ratio in the ACP group was 1.2:1, while the ratio in the PCP group was 1.8:1 (*P*=0.046). The ACP group had a bimodal distribution of age, while the PCP group showed a normal distribution of age (**Figure 2**). The age of onset in the ACP group was significantly lower compared to the PCP group (*P*<0.01) (**Table 2**).

**Table 2:** Demographic and clinical characteristics at baseline.

Variables	CP total (n=742)	ACP group (n=617)	PCP group (n=125)	*P*-value (ACP vs. PCP)
Age (years)	21 (7, 44)	14 (6, 39)	41.94 (14.13)	<0.01
Sex (Male/Female)	421/321	340/277	81/44	0.046
Tumor mass effects ^a^ (Yes/No)	548/194	446/171	102/23	0.03
Resection (GTR/partial)	609/133	506/111	103/22	0.92
Surgical method (Transsphenoidal/Transcranial)	115/627	89/528	26/99	0.07
Tumor position (intrasellar/suprasellar/both)	17/418/307	16/316/285	1/102/22	<0.01
Tumor volume ^b^ (cm^3^)	11.3 (5.5, 21.0)	12.3 (6.0, 21.3)	7.8 (3.7, 15.8)	<0.001
Average diameter of tumor (cm)	2.8 (2.3, 3.5)	3.0 (2.3, 3.5)	2.5 (2.0, 3.2)	<0.001
Follow-up (months)	15 (4, 27)	15 (4, 29)	13 (3, 24)	0.30
Recurrence ^c^ or Progression ^d^ (n, %)	160, 21.6%	137, 22.2%	23, 18.4%	0.35
Recurrence (n, %)	120, 19.7% (120/609)	103, 20.4% (103/506)	17, 16.5% (17/103)	0.37
Progression (n, %)	40, 30.1% (40/133)	34, 30.6% (34/111)	6, 27.3% (6/22)	0.75
*P*-value (Recurrence vs. Progression)	<0.01	0.02	0.24	–
Time of tumor recurrence/progression (months)	24 (15, 36)	24 (15, 36)	20 (12, 36)	0.37
Average diameter of recurrent/progressive tumor (cm)	2.8 (2.0, 3.4)	2.9 (2.0, 3.75)	2.5 (2.0, 3.0)	0.49
Re-operation (n, %)	87, 54.4% (87/160)	76, 55.5% (76/137)	11, 47.8% (11/23)	0.50
Radiotherapy (n, %)	21, 13.1% (21/160)	12, 8.8% (12/137)	9, 39.1% (9/23)	<0.01

a Tumor mass effects refers to that the patient has at least one of the following three symptoms: intracranial hypertension (headache or nausea), vision loss, and visual-field defect.

b Tumor volume = Length × Width × Height × 0.5.

c Recurrence: patients undergoing gross-total resection (GTR) had tumor markers again on postoperative MRI.

d Progression: an increase in residual tumor volume indicated by MRI during follow-up in patients undergoing partial resection.

The proportions of patients with headaches, impaired vision and visual field, vomiting, and other tumor mass effects before surgery were significantly higher in the PCP group than in the ACP group (81.6% vs. 72.3%, *P*=0.03). The ACP group showed significantly higher tumor volume and higher average tumor diameter compared with the PCP group (*P*<0.001). In addition, the proportion of patients with suprasellar tumors in the PCP group was significantly higher, while the proportions of patients with intrasellar or both intrasellar and suprasellar tumors were higher in the ACP group. There was no significant difference in the GTR/partial resection, surgical method, and follow-up time between the two groups (**Table 2**).

The median follow-up of all CP patients was 15 months, during which 21.6% of the patients experienced tumor recurrence or progression, with a median time of tumor recurrence/progression of 24 months and an average diameter of 2.8 cm. In all CP patients, the proportion of tumor recurrence in CP patients who underwent GTR was significantly lower than the proportion of tumor progression in patients who underwent partial resection at the end of follow-up (19.7% vs. 30.1%, *P*<0.01). The same difference was also observed in the ACP group (20.4% vs. 30.6%, *P*<0.02). The proportion of patients with recurrence/progression who underwent a second surgery was 54.4%. There was no significant difference in the follow-up time, recurrence/progression rate, time of tumor recurrence/progression, average diameter of tumor recurrence/progression, and re-operation rate between the ACP and PCP groups. However, the proportion of patients who received radiotherapy after recurrence or progression was significantly higher in the PCP group than in the ACP group (*P*<0.001) (**Table 2**).

### 3.2 Characteristics of HPD before and after surgery

Before surgery, the proportions of patients with DI (36.8% vs. 21.4%) and hyperprolactinemia (45.6% vs. 33.1%) were significantly higher in the PCP group than in the ACP group (both *P*<0.01). The proportion of patients with HPA dysfunction was significantly higher in the ACP group than in the PCP group (21.1% vs. 12.8%, *P*=0.03). There was no significant difference in the proportion of patients with other HPDs before surgery between the two groups. Of 742 CP patients, 22.5% had no HPD before surgery. The 10 disorders in the NEDS with the highest to the lowest proportion of patients were: HPG (37.8%), hyperprolactinemia (35.2%), HPT (24.9%), DI (24.0%), GHD (23.0%), HPA (19.7%), HO (19.1%), sleep disorder (4.9%), PCA (2.7%), ABTR (0.9%) (**Table 3**).

**Table 3:** Characteristics of HPD in CP patients before and after surgery.

Variables	CP total (n=742)			ACP group (n=617)			PCP group (n=125)			Pre-operation *P*-value (ACP vs. PCP)	Post-operation *P*-value (ACP vs. PCP)
	Pre-operation	Post-operation	*P*-value	Pre-operation	Post-operation	*P*-value	Pre-operation	Post-operation	*P*-value		
Hypothalamic dysfunction											
ABTR (n, %)	7, 0.9%	11, 1.5%	0.34	6, 1.0%	10, 1.6%	0.31	1, 0.8%	1, 0.8%	1.00	0.86	0.77
HO (n, %)	142, 19.1%	191, 25.7%	<0.01	118, 19.1%	163, 26.4%	<0.01	24, 19.2%	28, 22.4%	0.53	0.98	0.35
Sleep disorder (n, %)	36, 4.9%	39, 5.3%	0.72	28, 4.5%	30, 4.9%	0.79	8, 6.4%	9, 7.2%	0.80	0.38	0.29
PCA (n, %)	20, 2.7%	25, 3.4%	0.45	16, 2.6%	19, 3.1%	0.61	4, 3.2%	6, 4.8%	0.52	0.94	0.48
DI (n, %)	178, 24.0%	446, 60.1%	<0.01	132, 21.4%	376, 60.9%	<0.01	46, 36.8%	70, 56.0%	<0.01	<0.01	0.30
Pituitary dysfunction											
HPA (n, %)	146, 19.7%	561, 75.6%	<0.01	130, 21.1%	480, 77.8%	<0.01	16, 12.8%	81, 64.8%	<0.01	0.03	0.02
HPT (n, %)	184, 24.8%	621, 83.7%	<0.01	147, 23.8%	524, 84.9%	<0.01	37, 29.6%	97, 77.6%	<0.01	0.17	0.04
GHD (n, %)	171, 23.0%	414, 63.4%^a^	<0.01	143, 23.2%	367, 67.6%^b^	<0.01	28, 22.4%	47, 42.7%^c^	<0.01	0.85	<0.01
HPG (n, %)	172, 37.8%^d^	367, 76.6%^e^	<0.01	128, 38.1%^f^	278, 77.4%^g^	<0.01	44, 37.0%^h^	89, 74.2%^i^	<0.01	0.83	0.79
Hyperprolactinemia (n, %)	261, 35.2%	255, 34.4%	0.74	204, 33.1%	196, 31.8%	0.63	57, 45.6%	59, 47.2%	0.80	<0.01	0.01
Total HPD (n, %)	575, 77.5%	719, 96.9%	<0.01	477, 77.3%	600, 97.2%	<0.01	98, 78.4%	119, 95.2%	<0.01	0.79	0.25

a 89 patients were not evaluated for GH-IGF-1 axis.

b 74 patients were not evaluated for GH-IGF-1 axis.

c 15 patients were not evaluated for GH-IGF-1 axis.

d 287 children (males < 14 years, females < 13 years) were not evaluated for HPG.

e 263 children (males < 14 years, females < 13 years) were not evaluated for HPG.

f 281 children (males < 14 years, females < 13 years) were not evaluated for HPG.

g 258 children (males < 14 years, females < 13 years) were not evaluated for HPG.

h 6 children (males < 14 years, females < 13 years) were not evaluated for HPG.

i 5 children (males < 14 years, females < 13 years) were not evaluated for HPG.

After surgery, the proportions of patients with impaired HPA, HPT, and GHD in the ACP group were significantly higher than those in the PCP group (77.8% vs. 64.8%, *P*=0.02; 84.9% vs. 77.6%, *P*=0.04; 67.6% vs. 42.7%, *P*<0.01, respectively). The proportion of hyperprolactinemia was significantly higher in the PCP group than in the ACP group (47.2% vs. 31.8%, *P*=0.01) (**Table 3**).

### 3.3 Comparison of HPD in CP patients before and after surgery

**Figure 3:**
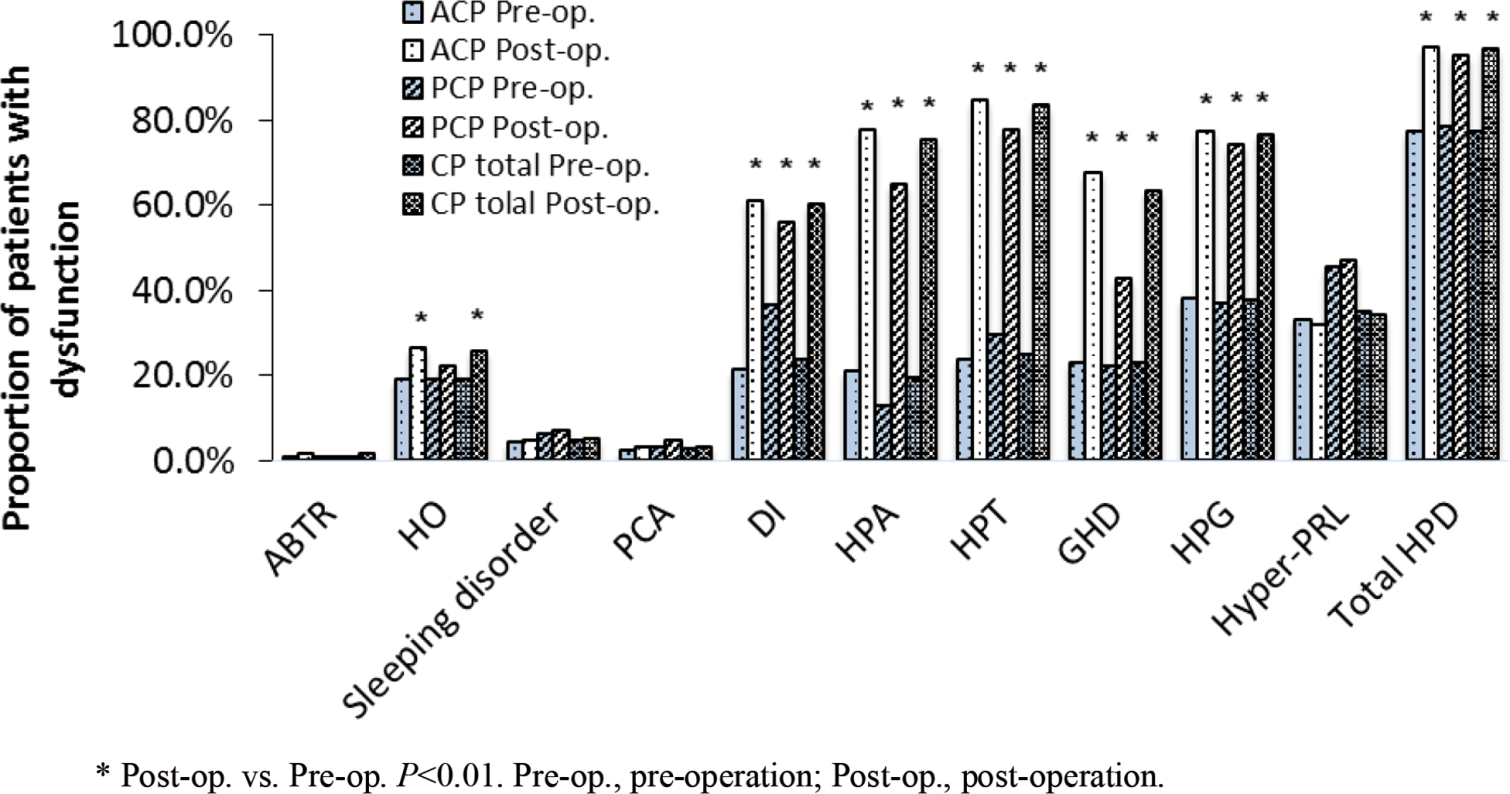
The proportion of patients with dysfunction in each nueroendocrine disorder before and after surgery.

Next, we compared the characteristics of HPD of all CP patients before and after surgery. In both the ACP and PCP groups, the proportions of patients with impaired HPA, HPT, HPG, GHD, and DI were significantly higher post-operatively compared to pre-operatively (*P*<0.01). In the ACP group, the proportion of patients with HO was significantly increased after surgery (26.4% vs. 19.1%, *P*<0.01), but no significant difference was observed in the PCP group (**Figure 3**). Among the 10 disorders in the NEDS, the proportion of patients with at least one disorder was increased in both groups after surgery (ACP: 97.2% vs. 77.3% before surgery, *P*<0.01; PCP: 95.2% vs. 78.4% before surgery, *P*<0.01). These results suggest that surgical treatment significantly increased the proportions of patients with HPD, especially adenohypophyseal dysfunction and DI.

### 3.4 Comparison of the NEDS score before and after surgery

We further analyzed the NEDS score of all CP patients, the ACP group, and the PCP group before and after surgery. Of all CP patients, 71.8% had a preoperative score of 0–2 and 22.5% had normal endocrine function (score 0). At the end of follow-up, 69.9% had a postoperative score of 3–5 and only 3.1% had normal endocrine function (score 0). This trend was also observed in the ACP and PCP groups. Surgical treatment significantly aggravated HPD in CP patients (*P*<0.001), especially in the ACP group (*P*=0.003) (**Table 4**).

**Table 4:** Comparison of the NEDS score before and after surgery.

NEDS score	CP total (n=742) (*P*<0.001)		ACP group (n=617) (*P*<0.001)		PCP group (n=125) (*P*<0.001)	
	Pre-operation	Post-operation	Pre-operation	Post-operation	Pre-operation	Post-operation
0	167, 22.5%	23, 3.1%	140, 22.7%	17, 2.8%	27, 21.6%	6, 4.8%
1	198, 26.7%	57, 7.7%	171, 27.7%	47, 7.6%	27, 21.6%	10, 8.0%
2	168, 22.6%	71, 9.6%	147, 23.8%	54, 8.8%	21, 16.8%	17, 13.6%
3	109, 14.7%	130, 17.5%	88, 14.3%	114, 18.5%	21, 16.8%	16, 12.8%
4	59, 8.0%	213, 28.7%	44, 7.1%	179, 29.0%	15, 12.0%	34, 27.2%
5	27, 3.6%	176, 23.7%	16, 2.6%	149, 24.1%	11, 8.8%	27, 21.6%
6	12, 1.6%	58, 7.8%	9, 1.5%	46, 7.5%	3, 2.4%	12, 9.6%
7	2, 0.3%	11, 1.5%	2, 0.3%	9, 1.5%	0	2, 1.6%
8	0	3, 0.4%	0	2, 0.3%	0	1, 0.8%
0-2	71.8%	20.4%	74.2%	19.2%	60%	26.4%
3-5	26.3%	69.9%	24.0%	71.6%	37.6%	61.6%

The Wilcoxon signed-rank test was used for intra-group comparisons of the NEDS score before and after surgery in each group. The Mann-Whitney U test was used to compare the degree of postoperative HPD between the ACP and PCP groups, P=0.003.

We further analyzed changes in the NEDS score in different groups of patients (**Table 5**). The proportions of patients with a decreased NEDS score after surgery did not significantly differ between the ACP and PCP groups (*P*=0.16). The proportions of patients with no change in the NEDS score were 10.2% and 19.2% in the ACP and PCP groups, respectively (*P*<0.01). The proportions of patients with an increased NEDS score after surgery was significantly higher in the ACP group than in the PCP group (*P*=0.001).

**Table 5:** Changes in the NEDS score in CP patients.

Changes in the NEDS score ^a^	CP total	ACP group	PCP group	*P*-value (ACP vs. PCP)
Alleviation	65, 8.8%	50, 8.1%	15,12.0%	0.160
-4	1,0.1%	1,0.2%	0	–
-3	3, 0.4%	2, 0.3%	1, 0.8%	-
-2	11, 1.5%	8, 1.3%	3, 2.4%	–
-1	50,6.7%	39, 6.3%	11, 8.8%	-
Unchanged 0	87, 11.7%	63, 10.2%	24, 19.2%	0.004
Aggravation	590, 79.5%	504, 81.7%	86, 68.8%	0.001
1	148, 19.9%	127, 20.6%	21, 16.8%	-
2	149, 20.1%	121, 19.6%	28, 22.4%	-
3	141, 19.0%	122, 19.8%	19, 15.2%	-
4	109, 14.7%	95, 15.4%	14, 11.2%	-
5	28, 3.8%	25, 4.1%	3, 2.4%	-
6	13, 1.8%	13, 2.1%	0	-
7	2, 0.3%	1, 0.2%	1, 0.8%	-

a Changes in NEDS score = Postoperative NEDS score – Preoperative NEDS score. –4 to –1: the number of disorders decreased after surgery; 0: no change; 1 to 7: the number of disorders increased after surgery.

### 3.5 The risk factors for aggravation of HPD in CP patients after surgery

Surgical treatment is the preferable option for effectively removing the intracranial mass in CP patients, but it inevitably aggravates HPD in this population. Here, we performed univariate and multivariate logistic regression analyses to identify the risk factors for postoperative aggravation of HFD in the CPs. The independent variables were sex, age of onset, tumor mass effects, surgical method, resection (GTR/partial), pathological type, recurrence or progression, average diameter of tumor, tumor location, and pre-operative NEDS. Whether the postoperative NEDS score increased or not was defined as the dependent variable. The univariate logistic regression analysis showed that older age at onset, tumor recurrence or progression, transcranial surgery, ACP type, partial resection, suprasellar origin and growth, and low preoperative NEDS score increased the risk of HPD in patients after surgery (*P*<0.20). After adjustment for potential confounding factors, including sex, tumor mass effects, and tumor diameter, we found that older age of onset (odds ratio (OR)=1.01; 95% confidence interval (CI): 1.00–1.03; *P*=0.04), tumor recurrence or progression (OR=2.21; 95% CI: 1.26–3.88; *P<*0.01), ACP type (OR=2.02; 95% CI: 1.17–3.49; *P*=0.01), and low preoperative NEDS score (OR=0.53; 95% CI: 0.46–0.61; *P*<0.001) remained significantly correlated with increased postoperative NEDS score. In addition, we investigated the impact of tumor recurrence or progression on the aggravation of HFD in CP patients who underwent GTR versus those who underwent partial resection. Multivariate logistic regression analyses showed that recurrence was an independent risk factor for an increased NEDS score in CP patients (OR=2.15; 95% CI: 1.13–4.08; P=0.02) (**Table 6**).

**Table 6:** The risk factors for the aggravation of HPD in CP patients after surgery.

Parameters	Univariate		Multivariate	
	OR (95% CI)	*P*-value	OR (95% CI)	*P*-value
Sex (Male/Female)	0.91 (0.64, 1.31)	0.61	1.04 (0.69, 1.56)	0.86
Age of onset (Years)	1.00 (0.99, 1.01)	0.13	1.01 (1.00, 1.03)	0.04
Tumor mass effects (Yes/No)	0.89 (0.59, 1.34)	0.57	0.86 (0.54, 1.37)	0.53
Surgical method (Transsphenoidal/Transcranial)	1.63 (1.04, 2.56)	0.04	1.49 (0.85, 2.60)	0.17
Resection (GTR/partial)	0.67 (0.43,1.03)	0.07	0.73 (0.44, 1.20)	0.22
Classification (ACP/PCP)	2.02 (1.32, 3.11)	<0.01	2.02 (1.17, 3.49)	0.01
Recurrence or Progression (Yes/No)	2.20 (1.31, 3.69)	<0.01	2.21 (1.26, 3.88)	<0.01
Recurrence (Yes/No)	2.24 (1.24, 4.05)	<0.01	2.15 (1.13, 4.08)	0.02
Progression (Yes/No)	1.83 (1.09, 3.06)	0.02	1.56 (0.85, 2.85)	0.15
Average diameter of tumor (cm)	1.12 (0.93, 1.36)	0.24	1.21 (0.96, 1.53)	0.11
Tumor location		0.08		0.29
Suprasellar vs. Intrasellar	3.00 (1.11, 8.14)	0.03	2.66 (0.79, 8.95)	0.12
Intrasellar and suprasellar vs. Intrasellar	2.56 (0.94, 6.98)	0.07	2.41 (0.72, 8.10)	0.16
Pre-operative NEDS	0.54 (0.48, 0.62)	<0.001	0.53 (0.46, 0.61)	<0.001

## 4 Discussion

In this study, the proportion of male to female subjects was 1.3:1, which was in line with the study by Feng et al. (18), but different from the findings by Müller et al., which showed no sex difference in the incidence of CP (19). This difference may have been because only patients undergoing surgery were enrolled in our study.

The main clinical manifestations of CP are tumor mass effects and HPD. In this study, more patients in the PCP group presented with tumor mass effects before surgery compared with the ACP group. The proportions of CDI and hyperprolactinemia were also higher in the PCP group. The proportion of HPA dysfunction was significantly higher in the ACP group than in the PCP group. Meanwhile, patients with ACP showed higher tumor volume and tumor diameter before surgery. We found that ACPs mostly originated in the sellar region, while PCPs mostly originated in the suprasellar cisterna. Despite the smaller tumor volume of PCP versus ACP, the proportion of tumor mass effects was higher and hypothalamic dysfunction was more common in cases with PCP due to the suprasellar origin. A higher proportion of ACP cases had an intrasellar origin, resulting in a higher prevalence of adenohypophyseal dysfunction compared to patients with PCP. Our presumption is that the location of tumor origin, rather than its volume or diameter, is more closely related to hypothalamic dysfunction and tumor mass effects in patients with CP (20, 21).

The recurrence rate following GTR ranges between 8% and 46% (22, 23) In this study, we found that the tumor progression rate in patients undergoing partial resection was higher than the recurrence rate in patients undergoing GTR in both the ACP and PCP groups. However, there was no significant difference in tumor recurrence and progression rates between the ACP and PCP groups. Therefore, when a CP does not involve the hypothalamus, surgery with the goal of GTR is often preferred (23). It is worth noting that a significantly higher proportion of patients received radiotherapy after tumor recurrence/progression in the PCP group than in the ACP group (*P*<0.01), which may be associated with two factors. Firstly, the proportion of children in the ACP group was significantly higher than that of the PCP group, and secondly, radiation therapy may have more severe adverse effects on children than on adults, particularly in terms of growth and development. Previous studies have shown that radiotherapy may also lead to irreversible and severe damage to the hearing, vision, and cognitive functions of pediatric patients (24). Although newly developed pencil beam proton therapy has significantly reduced tissue damage and cognitive impairment compared with traditional photon or gamma-ray therapy (8), there is still no consensus on whether proton therapy should be recommended for children with postoperative residual tumors or tumor recurrence (25). Limited surgery and radiotherapy are recommended for tumors that have invaded the hypothalamus in adults, depending on factors such as tumor extent, recurrence, and the relationship between tumor and the stalk or optic nerve, as well as the patient’s age (26). However, there is ongoing debate in the literature about whether radiation contributes to malignant transformation (27, 28). Our center reported seven cases of malignant transformation from benign CP, all of which were in children or adolescents (average age of 22 years) who had received radiotherapy. Although no differences in pathological subtypes were observed at onset, the association with radiotherapy raises concerns about its potential role in the transformation process. Therefore, patients with CP, especially those at a younger age, should be fully evaluated and informed of potential risks before radiotherapy (29).

In this study, only 22.5% of the patients showed no HPD before surgery, and the proportions of DI and adenohypophyseal dysfunction before surgery were consistent with previous data (3–5). Previous evidence has revealed that most patients have severe HPD when CP is diagnosed, such as GHD (35–95%), FSH and LH deficiency (38–82%), ACTH deficiency (21–62%), TSH deficiency (21–42%), and DI (6–38%) (30). Few large-scale clinical studies have reported the characteristics of hypothalamus syndrome in patients with CP, possibly because the clinical evaluation of sleep disorder and PCA, two major manifestations of hypothalamus syndrome, is difficult and requires multidisciplinary collaboration between Departments of Psychology, Endocrinology, Pediatrics, and Neurosurgery, as well as the sleep center (4). In this study, only patients with clinical manifestations of sleep problems or behavioral, psychological, and cognitive abnormalities were given sleep and PCA questionnaires suitable for their age. Therefore, the occurrence rate of sleep disorders and PCA in this study may be underestimated. Further investigations on the effects of hypothalamic syndrome on the prognosis of CP are needed (31).

The proportion of patients with HPD was significantly increased after surgery in both the ACP and PCP groups, especially in DI and adenohypophyseal dysfunction (except for hyperprolactinemia), suggesting that surgery effectively alleviated the tumor mass effect, but aggravated the degree of HPD in both the ACP and PCP groups. Recent studies have confirmed that, unlike pituitary adenomas, surgical treatment of CP rarely restores preexisting hormone deficiency. With the intervention of secondary surgery and radiotherapy, the incidence of long-term endocrine hormone deficiency in CP patients is significantly increased (32).

In this study, the proportion of patients with HO in the ACP group was significantly increased after surgery (*P*<0.01), but not in the PCP group, probably due to different distributions of age between the two groups. Previous studies have also shown that the postoperative incidence of overweight or obese cases is higher in children with CP than in adults (48–70% vs. 31–59%) (33, 34). Postoperative weight gain may be related to invasive tumor treatment, which damages the paraventricular nucleus and the suprachiasmatic nucleus, leads to loss of satiety, and inhibits the interaction of leptin, insulin, and intestinal hormones (35, 36). It is worth noting that increased body fat content will inhibit the secretion of gonadotropin and affect the function of the HPG axis in CP patients (37). It was reported that hypothalamic involvement has a statistically significant negative effect on the 20-year overall survival in children with CP, while the degree of surgical resection had no effect on 20-year progression-free survival (38). These findings support the concept that attempts should be made to preserve the stalk and hypothalamus if GTR can be achieved, in order to reduce the risk of postoperative endocrinopathy (25).

Previous studies on the endocrine function of patients with CP have mainly focused on a single endocrine function axis. In this study, the NEDS was used to quantitatively evaluate the global hypothalamic-pituitary-target gland axis in patients with CP. While the NEDS has not previously been proposed, the diagnostic criteria for each index in the NEDS are quoted from recognized guidelines. We observed that patients with ACP had a higher risk of exacerbation of HPD after surgery compared to the PCP group, indicating that the NEDS can be a valuable tool for clinicians to quantitatively evaluate and gain a more comprehensive understanding of the neuroendocrine function of patients with CP, through the accumulation of data on various hypothalamic-pituitary functions. In addition, the presence and severity of hypothalamic syndrome in children with CP can be assessed using clinical (39) or radiological scales, such as the Muller radiological score (40).

The identification of risk factors for poor neuroendocrine outcomes in patients with CP may facilitate early prevention and intervention of HPD in these patients. Hoffmann et al. reported that the course of disease in children with CP before diagnosis was positively correlated with age and proportion of impaired endocrine function, but not with tumor size at diagnosis, body mass index, GTR, and hypothalamus involvement (41). In this study, we found that postoperative aggravation of HPD was related to older age of onset, tumor recurrence or progression, ACP type, and low preoperative NEDS score, but not related to sex, tumor size, tumor mass effects, transcranial or transsphenoidal operation, tumor location, and GTR/partial resection. Notably, the risk of postoperative aggravation of HPD in the ACP group was two times higher than that of the PCP group, indicating that patients with different CP subtypes may have different endocrine function outcomes. In addition, recurrence was a risk factor for the aggravation of HPD in patients after GTR. However, we did not observe a significant effect on the NEDS score in patients presenting with tumor progression after partial resection. CP patients undergoing partial resection often have large tumor volumes, invasion of surrounding tissues, and a high proportion of transcranial surgery (23, 42). These factors result in a higher NEDS score caused by the tumor itself before surgery, which may explain the lack of significant increases in the NEDS score after tumor progression compared to before surgery.

## 5 Conclusion

Patients with ACP are more likely to be complicated with adenohypopituitarism, while those with PCP are more prone to develop hypothalamic dysfunction (i.e., DI and hyperprolactinemia), possibly because more ACP originates in the sellar region and more PCP originates in the suprasellar region. Although surgery is the preferred method for treating CP, it also exacerbates HPD in both ACP and PCP cases. Moreover, neuroendocrine function is more severely damaged in patients with ACP after surgery. Older age at onset, tumor recurrence or progression, and ACP type are risk factors for postoperative aggravation of HPD in patients with CP. In addition, patients with a low NEDS score before surgery were more likely to have a high postoperative score. Therefore, patients with CP who exhibit these characteristics should get appropriate hormone replacement and functional rehabilitation treatment, as well as particular attention to postoperative neuroendocrine function.

## Data availability statement

The raw data supporting the conclusions of this article will be made available by the authors, without undue reservation.

## Ethics statement

Data analysis was performed retrospectively, with all procedures and analysis approved by the local ethics committee according to the Declaration of Helsinki (IRB of Beijing Tiantan Hospital, Capital Medical University, KY2022-024-01). Written informed consent to participate in this study was provided by the participants’ legal guardian/next of kin.

## Author contributions

LZ and YZ designed the study. LP and YL collected and analyzed the data. SG, CL, PL and MN were the main surgeons responsible for CP operations. YG wrote the paper. All authors contributed to the article and approved the submitted version.
